# Irreproducibility in searches of scientific literature: A comparative analysis

**DOI:** 10.1002/ece3.8154

**Published:** 2021-10-14

**Authors:** Gábor Pozsgai, Gábor L. Lövei, Liette Vasseur, Geoff Gurr, Péter Batáry, János Korponai, Nick A. Littlewood, Jian Liu, Arnold Móra, John Obrycki, Olivia Reynolds, Jenni A. Stockan, Heather VanVolkenburg, Jie Zhang, Wenwu Zhou, Minsheng You

**Affiliations:** ^1^ State Key Laboratory of Ecological Pest Control for Fujian and Taiwan Crops Institute of Applied Ecology Fujian Agriculture and Forestry University Fuzhou China; ^2^ Joint International Research Laboratory of Ecological Pest Control Ministry of Education Fuzhou China; ^3^ Department of Agroecology Flakkebjerg Research Centre Aarhus University Slagelse Denmark; ^4^ UNESCO Chair on Community Sustainability: From Local to Global Department of Biological Science Brock University St. Catharines ON Canada; ^5^ Graham Centre for Agricultural Innovation (Charles Sturt University and NSW Department of Primary Industries) Charles Sturt University Orange NSW Australia; ^6^ Agroecology University of Goettingen Goettingen Germany; ^7^ “Lendület” Landscape and Conservation Ecology Institute of Ecology and Botany Centre for Ecological Research Vácrátót Hungary; ^8^ Department of Biology Savaria Campus Eötvös Loránd University Szombathely Hungary; ^9^ Department of Environmental Sciences Sapientia Hungarian University of Transylvania Cluj‐Napoca Romania; ^10^ Department of Water Supply and Sewerage Faculty of Water Science National University of Public Service Baja Hungary; ^11^ Aquatic Ecological Institute Centre for Ecological Research Budapest Hungary; ^12^ Department of Zoology University of Cambridge Cambridge UK; ^13^ Department of Rural Land Use SRUC Aberdeen UK; ^14^ Department of Hydrobiology Institute of Biology University of Pécs Pécs Hungary; ^15^ University of Kentucky Lexington USA; ^16^ Cesar Parkville VIC Australia; ^17^ Biosecurity and Food Safety NSW Department of Primary Industries Narellan NSW Australia; ^18^ Department of Ecological Sciences The James Hutton Institute Aberdeen UK; ^19^ State Key Laboratory of Rice Biology Key Laboratory of Molecular Biology of Crop Pathogens and Insects Ministry of Agriculture Zhejiang University Hangzhou China

**Keywords:** database, evidence synthesis methods, information retrieval, repeatability, reproducibility, search engine, search location

## Abstract

1. Repeatability is the cornerstone of science, and it is particularly important for systematic reviews. However, little is known on how researchers’ choice of database, and search platform influence the repeatability of systematic reviews. Here, we aim to unveil how the computer environment and the location where the search was initiated from influence hit results.
2. We present a comparative analysis of time‐synchronized searches at different institutional locations in the world and evaluate the consistency of hits obtained within each of the search terms using different search platforms.
3. We revealed a large variation among search platforms and showed that PubMed and Scopus returned consistent results to identical search strings from different locations. Google Scholar and Web of Science's Core Collection varied substantially both in the number of returned hits and in the list of individual articles depending on the search location and computing environment. Inconsistency in Web of Science results has most likely emerged from the different licensing packages at different institutions.
4. To maintain scientific integrity and consistency, especially in systematic reviews, action is needed from both the scientific community and scientific search platforms to increase search consistency. Researchers are encouraged to report the search location and the databases used for systematic reviews, and database providers should make search algorithms transparent and revise access rules to titles behind paywalls. Additional options for increasing the repeatability and transparency of systematic reviews are storing both search metadata and hit results in open repositories and using Application Programming Interfaces (APIs) to retrieve standardized, machine‐readable search metadata.

## Graphical abstract

The consistency level with which scientific search platforms return hits for the same search initiated at different locations and from different computing environments was investigated. Scopus and PubMed returned the same results regardless of the location or computing environment but Web of Science and Google Scholar varied substantially. Since this is a crucial issue for the repeatability of systematic reviews, action should be taken to improve the within‐database consistency in the returned search hits.

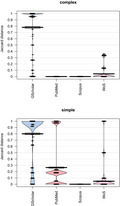

## 1 INTRODUCTION

Scientific literature is rapidly expanding (Bornmann & Mutz, 2015), making it impossible to track new discoveries by focusing only on the primary literature (Landhuis, 2016; Pain, 2016). Thus the importance of systematic reviews continues to increase (Gurevitch et al., 2018). Whereas in narrative reviews the literature inclusion/exclusion criteria and the evaluation processes are often ambiguous, and the allocation of the level of importance devoted to individual studies are unclear (Clarke & Horton, 2001), systematic reviews are supposed to be highly transparent and repeatable. These are especially important when the available body of evidence is controversial. With the advent and rapid development of Internet‐based databases and search engines (together termed as “search platforms” or “platforms” hereafter), the role of narrative reviews is now being surpassed by new, quantitative methods of evidence synthesis (Garg et al., 2008; Ioannidis, 2016).

Knowledge synthesis in evidence‐based methods is a highly structured process with standard, well‐defined steps, for which articulate guidance is available in several fields, including ecology (Pullin & Stewart, 2006) and medical science (Haddaway et al., 2020; Higgins et al., 2019). The two most important principles are universal: transparency and repeatability. During the process, all the steps taken and decisions made have to be documented in detail, which is a crucial condition to repeatability. Repeatability, as a core requirement in these activities, crucially depends on reliable databases (Gusenbauer & Haddaway, 2019). Large scientific databases and search platforms, such as PubMed, Web of Science and Scopus, are essential in this process. They have been primary electronic search platforms for scientists since 1997 with the inauguration of PubMed (Falagas et al., 2007). Today, nearly all scientists working on various forms of evidence‐based synthesis use these platforms to find relevant papers as the basis for further analysis.

An important consideration in the whole process is that the evidence base must be solid: a given search string on the same database/search platform should generate identical results, independent of search locations (i.e. institutional background), provided the searches are running at the same time. If this assumption were violated, it would have serious consequences for the reliability and repeatability of the data and papers selected for a systematic review. Therefore, there is a need to know what variables affect consistency of searches in each database and define which database or engine search is going to be used for obtaining the data to be synthesized.

The most commonly used search platforms, Google Scholar, PubMed, Scopus, and Web of Science, are known to yield different results for the same search strings (Boeker et al., 2013; Gavel & Iselid, 2008; Gusenbauer & Haddaway, 2019). The reasons are simple; PubMed, Scopus, and Web of Science use different background databases, whilst Google Scholar, without having a well‐defined background database, uses crawl robots to search sites on the Internet. Yet, knowledge of the consistency *within* each search platform in relation to the location (i.e. institutional server) where the search is requested from, software environment, or computer configuration remain surprisingly limited (but see Gusenbauer & Haddaway, 2019 for location consistencies of scientific search platforms and Cooper et al., 2021 for geographic varions in Google search results). Since the search histories of users may be stored in the browsers’ cache and considered by the scientific search platforms, repeated and identical searches may result in different outcomes.

During a recent systematic review in ecology, we accidentally discovered that a multilocus search performed on 1 February 2018, using an identical search string in Web of Science Core Collection, produced radically different number of hits at different institutions at Hangzhou and Fuzhou, in China and in Denmark (2,394, 1,571, and 7,447, respectively). This triggered us to systematically explore this issue.

Since there is no known study comparing the consistency of returned papers over successive identical searches using several platforms in one machine, we examined the way databases and search engines deliver results and decided to systematically explore the inconsistencies found. Our study aimed to evaluate the consistency of search platforms by comparing the outcomes from identical search strings run on different computers in twelve localities across the world, with various software backgrounds.

To investigate the repeatability of scientific searches in four of the major databases and search engines, Web of Science, Scopus, PubMed, and Google Scholar, we generated search strings with ecological terms and two complexity levels, ran standardized searches from various institutions in the world, within a limited timeframe, and tested within‐platform discrepancies in hit results.

## 2 MATERIALS AND METHODS

### 2.1 Queried databases

Three major scientific search platforms, PubMed, Scopus, and Web of Science, and Google Scholar, were used in this study. Although Google Scholar is markedly different from the other three traditionally used platforms, both in business politics and search methods (Falagas et al., 2007; Jacsó, 2008), the increasing use of this search engine (Haddaway et al., 2015) justifies its inclusion in the study. In this manuscript, we are using the term “search platforms” to include all PubMed and Scopus which operate on a single database, Web of Science, which is a collection of databases, and Google Scholar which has no database. The main differences between these platforms are discussed below and have also been catalogued and reviewed by Falagas et al. (2007).

PubMed (https://www.ncbi.nlm.nih.gov/pubmed) is a freely available scientific database, focusing mostly on biomedical literature, which holds ca. 30 million citations covering a variety of aspects of life sciences (https://pubmed.ncbi.nlm.nih.gov/about/, accessed 15/11/2020). It was developed and is being maintained by the National Center for Biotechnology Information.

Scopus, is a database, currently owned by the Elsevier group. It contains bibliographic data of over 1.4 billion publications dating back to 1970. It indexes ca. 70 million items and 22,800 journals from over 5,000 publishers (https://www.elsevier.com/solutions/scopus/how‐scopus‐works/content, accessed: 15/11/2020).

Web of Science (https://webofknowledge.com) is the oldest scientific search platform, owned by the Clarivate Analytics (previously Thomson Reuters). Web of Science, running under its current name since 1997, is the successor of the first scientific citation database, the Current Contents/Science Citation Index, which was launched in 1964. Currently it consists of several databases, including Zoological Records, CABI Abstracts, and a number of other, formerly independent ones. It indexes 34,586 journals, books, and proceedings, and, as of the last update, on 15/11/2020, it covers 174 million records altogether and over 79 million in its Core Collection (https://clarivate.libguides.com/webofscienceplatform/coverage). Although in this study we queried only the Core Collection, the search system is unlikely to work differently for the other components of Web of Science. Therefore, we refer only to “Web of Science” as an inclusive term throughout the article.

Google Scholar (https://scholar.google.com) is a free online tool, the sub‐site of the search corporation Google Inc., which is particularly designed for scholarly searches. Instead of having a background database, Google Scholar uses a search engine with “crawler robots” to find relevant result on the World Wide Web. Whilst Google Scholar has been often criticized for not sharing its search algorithms, for its untraceable way of ordering search hits and for the inclusion of material from non‐scholarly sources in its research hits (Jacsó, 2005, 2008; Noruzi, 2005), it has been playing an increasing role in daily lives of scientists since its launch in 2004 (Haddaway et al., 2015; Halevi et al., 2017). It is also estimated to include 160 million individual scientific publications in 2014 (Orduna‐Malea et al., 2015), providing a high coverage in several scientific areas (Larsen & von Ins, 2010 and references therein). It is also the fastest growing resource for scientific literature (Gusenbauer, 2019). Its usefulness, however, for systematic reviews and meta‐analyses has been debated (Boeker et al., 2013; Jacsó, 2005, 2008).

### 2.2 Web searches

In our pilot search, we queried Google Scholar with the keyword expression “systematic review” AND “ecology” on 7 November 2018, from a server based in Hong Kong, to investigate researchers’ attitude to report information valuable for repeatability. Sites were restricted to sciencemag.org, nature.com, and wiley.com. Hits were sorted from the newly published to older, and the twenty first papers were examined (Appendix S1). We confirmed whether papers were using multiple search platforms and whether the exact time (by day) and location of the search were reported.

In order to investigate the repeatability of scientific searches in the four major search platforms, we generated keyword expressions (search strings) with two complexity levels using keywords that focused on an ecological topic and ran standardized searches from various institutions in the world (see below), all within a limited timeframe.

Simple search strings contained only one main key phrase, without using logical (Boolean) operators, whereas complex ones contained both inclusion and exclusion criteria for additional, related, keywords and key phrases (i.e. two‐word expressions within quotation marks). In complex search strings, Boolean operators were also used. The simple keyword was “ecosystem services” while the complex one was “ecosystem service” AND “promoting” AND “crop” NOT “livestock”. Search language was set to English in every case, and only titles, abstracts, and keywords were searched. Since there is no option in Google Scholar to limit the search to titles, keywords, and abstracts, we used the default search in this case. Since different search platforms use slightly different expressions for the same query, exact search term formats were generated for each search (Table 1).

**TABLE 1:** Search strings for each keyword complexity and topic, adjusted according to the search platform

Platform	Complex search string	Simple search string
GScholar	"ecosystem service" + "promoting" + "crop" – "livestock"	"ecosystem services"
PubMed	"ecosystem service"[Title/Abstract] AND "promoting" AND "crop"[Title/Abstract] NOT "livestock"[Title/Abstract] AND "english"[Language]	"ecosystem services"[Title/Abstract] AND "english"[Language]
Scopus	TITLE‐ABS‐KEY ("ecosystem service" AND "promoting" AND "crop" AND NOT "livestock") AND (LIMIT‐TO (LANGUAGE, "English"))	TITLE‐ABS‐KEY ("ecosystem services") AND (LIMIT‐TO (LANGUAGE, "English"))
WoS	TS = ("ecosystem service" AND "promoting" AND "crop" NOT "livestock")	TS = ("ecosystem services")

Searches were conducted on one or two machines at each of the 12 institutions in Australia, Canada, China, Denmark, Germany, Hungary, UK, and the USA (Appendix S2), using three commonly used browsers (Mozilla Firefox, Internet Explorer, and Google Chrome). Searches were run manually (i.e. no APIs were used) according to strict protocols, which allowed standardization of search date, exact search term for every run, and the data recording procedure. Not all platforms were queried from every location: Google products are not available in China, and Scopus was not available at some institutions (Appendix S2). The original version of the protocol is provided in Appendix S3. The first run was conducted at 11:00 Australian Eastern Standard Time (01:00 GMT) on 13 April 2018 and the last search run at 18:16, Eastern Daylight Time (22:16 GMT, 13 April 2018). After each search run, the number of hits was recorded, and the bibliographic data of the first 20 articles were extracted and saved in a file format that the website offered (.csv,.txt). Once search combinations were completed, the browsers’ cache was emptied, to make sure the testers’ previous searches did not influence the results, and the process was repeated. At four locations (Flakkebjerg, Denmark; Fuzhou, China; St. Catharines, Canada; Orange, Australia), the searches were also repeated on two different computers. This resulted in 228, 132, 228, and 144 search runs for Web of Science, Scopus, PubMed, and Google Scholar, respectively.

Results were collected from each contributor, and bibliographic information was automatically extracted from the identically structured saved files using a loop in the R statistical software (R Core Team, 2012) and stored in a standardized MySQL database, allowing unique publications to be distinguished. If unique identifiers for individual articles were missing, authors, titles, or the combination of these were searched for, and uniqueness was double checked across the entire dataset. Saved data files with nonstandard structures were dealt with manually. All data cleaning and manipulations were done by R.

### 2.3 Statistical analysis

To investigate how consistent the number of resulting hits from each search string was for each of the search platforms, *average absolute deviation* (AAD, i.e. the absolute value of the difference of the actual value and the mean) was calculated and expressed as a proportion of the mean of each group (‘*average absolute deviation proportion*’, AADP, i.e. search term complexity, and search platform). AADP was calculated using the equation:

$$\text{AADP}=\frac{\left|e‐{\hat{e}}_{\mathrm{gr}}\right|}{{\hat{e}}_{\mathrm{gr}}},$$

where *e* was the number of hits from one particular search and ${\hat{e}}_{\mathrm{gr}}$ was the mean number of hits of pooled numbers from one topic and search term complexity combination and one search platform (e.g. complex ecological search expression queried using Scopus). This grouping was necessary because the number of hits substantially differed depending on these three factors. Since the aim of the study was not to compare the efficiency of different search platforms, this grouping did not interfere with our analysis.

The normality of the data and their homoscedasticity were tested using Kolmogorov‐Smirnoff test and the Breusch Pagan test, respectively. These tests confirmed that the distribution of AADPs did not follow normal distribution and neither were the variances of the residuals homogenous within each group. Indeed, the high number of zeroes resulted in a zero‐inflated, an unbalanced beta distribution, as suggested by the *descdist*() function in the *fitdistrplus* R package (Delignette‐Muller & Dutang, 2015), in the R programming environment (R Core Team, 2012).

AADP is expected to be zero in cases when search platforms consistently give the same number of hits within groups, regardless where the search is initiated from, browser used, or whether the cache was emptied or not. Therefore, one‐sided Wilcoxon signed rank tests were performed for the AADP values for each search platform within each group to test if they were significantly different from zero.

To address non‐normality, unequal variances and to control Type I error, the non‐parametric, Welch‐James's statistic with Approximate Degrees of Freedom (Welch ADF) was used to investigate the differences between search platform consistencies and to select the most influential factors driving these differences. This robust estimator uses trimmed means and Winsorized variances to avoid biases derived from heteroscedasticity. Bootstrapping was used to calculate empirical p‐values both for between group and pairwise comparisons (Keselman et al., 2008), with the help of *WelchADF* R package (Villacorta, 2018).

Additionally, average similarities of the first 20 papers within each of the search platform–keyword complexity groups were calculated based on binary matrices, in which rows corresponded to search runs from various institutions and computers, whilst columns contained individual papers (thus lines representing individual ‘paper communities’). Due to its suitability for using binary data (Boyce & Ellison, 2001), Jaccard distance measures were applied for dissimilarity calculations and a matrix of pairwise distances of separate search runs was created. Distance‐based redundancy analysis (dbRDA, *capscale()* function) was used with the same distance matrix to ordinate the resultant article collections in each search topic–keyword complexity group. Convex hulls of the points resulted from this ordination were then delimited for each search platform, and their areas were calculated. Since similarities between article collections resulted from searches with a platform giving consistently the same hits, regardless of search location, browser used, and cache content, should always be zero, the ideal size of these hulls would be also zero. Multivariate analysis was conducted using the *vegan* (Oksanen et al., 2010) R package.

## 3 RESULTS

Of the twenty selected systematic reviews in our pilot search, nine queried only Web of Science (potentially including its "sister databases") to find relevant publications. Only two reported the date when the search was performed, and none reported the search location/institutional server.

Our time‐synchronized, cross‐institution, and multilocation search exercise resulted in a large variation in the number of hits obtained using any of the search terms. Google Scholar generally yielded a greater number of hits than any other databases for all the locations (Table 2).

**TABLE 2:** Comparison of the mean numbers of hits (*SD*) resulting from simple versus complex search strings in the fields of ecology and medicine using different search platforms, different browsers, and cache handling

Platform	Browser	Cache	Number of hits of search strings in thousands	
			Simple	Complex
Google Scholar	Chrome	Full	1,157.188 ± 991.840	2.069 ± 1.663
		Cleaned	871.186 ± 1,065.303	1.595 ± 1.699
	Internet Explorer	Full	1,077.496 ± 1,018.818	1.945 ± 1.685
		Cleaned	862.614 ± 1,054.802	1.595 ± 1.699
	Firefox	Full	905.849 ± 1,026.956	1.945 ± 1.684
		Cleaned	985.978 ± 1,036.853	1.816 ± 1.693
PubMed	Chrome	Full	2.881 ± 0.001	0.006 ± 0
		Cleaned	2.881 ± 0.001	0.006 ± 0
	Internet Explorer	Full	2.881 ± 0.001	0.006 ± 0
		Cleaned	2.881 ± 0.001	0.006 ± 0
	Firefox	Full	2.881 ± 0.001	0.006 ± 0
		Cleaned	2.881 ± 0.001	0.006 ± 0
Scopus	Chrome	Full	19.912 ± 0	0.078 ± 0
		Cleaned	19.912 ± 0	0.078 ± 0
	Internet Explorer	Full	19.912 ± 0	0.078 ± 0
		Cleaned	19.912 ± 0	0.078 ± 0
	Firefox	Full	19.912 ± 0	0.078 ± 0
		Cleaned	19.912 ± 0	0.078 ± 0
Web of Science	Chrome	Full	17.295 ± 1.214	15 ± 0
		Cleaned	17.561 ± 0.798	15 ± 0
	Internet Explorer	Full	17.642 ± 0.740	15 ± 0
		Cleaned	17.587 ± 0.832	15 ± 0
	Firefox	Full	17.492 ± 0.967	14.9 ± 0.49
		Cleaned	17.370 ± 0.978	14.8 ± 0.55

The *average absolute deviation proportion*s (AADP, see *Materials and Methods*) of every database and search engine, except Scopus, significantly deviated from the ideal of zero (Table 3). PubMed and Web of Science were updated during the search window, at 17:00 GMT and 19:00 GMT, respectively. When the results from these platforms were split into two groups, before and after the time of the daily update, none of the AADPs from PubMed searches significantly differed from zero. In contrast, the results from Web of Science searches consistently showed a statistically significant deviation, indicating inconsistency in the number of returned hits by search location or host institution.

**TABLE 3:** Mean and standard deviations of recorded average absolute deviation proportions (AADP) for each investigated search platforms, separated by search topic and search expression complexity

Keyword Complexity	GScholar	PubMed	Scopus	WoS
Complex	85.319 ± 9.426	0.000 ± 0.000	0.000 ± 0.000	0.629 ± 1.964
Simple	98.107 ± 4.063	0.035 ± 0.000	0.000 ± 0.000	4.009 ± 3.459

Note

Values are shown in percentage.

**FIGURE 1:**
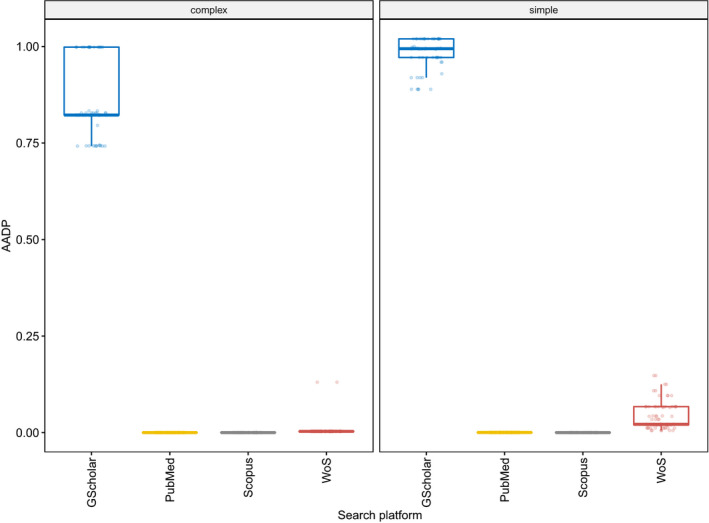
Boxplots showing average absolute deviation proportions (AADP) of hit numbers, grouped by searched platforms, and separated by keyword complexity (complex, simple). Median AADPs are indicated with a thick black line. Google Scholar is abbreviated as GScholar

The WelchADF test revealed significant differences in AADPs among groups (92.45% variance explained), with search platforms being the most important explanatory variable. Keyword complexity, platform, and their interacting effect were also significant predictors. The effect of browsers used was not significant, either alone or as a covariate of search platform choice. Emptying cache had no significant effect, either alone or as a covariant (Figure 1, Table 4, Appendix S4 and S5). Though not being a significant predictor overall, both browser and cache tended to influence the Google Scholar results. None of these influenced the search platforms with a background database. There were no differences in search results when Web of Science, PubMed, and Scopus were used on different machines at the same location, but Google Scholar sometimes produced different results.

**TABLE 4:** The results of the Welch‐James's statistic with Approximate Degrees of Freedom

	WJ statistics	Numerator DF	Denominator DF	*p*‐value
Search platform	**78,828.74**	**3**	**60.29**	**<.001**
Keyword complexity	**69.86**	**1**	**28.7**	**<.001**
Browser	0.01	2	19.46	.988
Cache	0.03	1	28.7	.841
Search platform:Keyword complexity	**76,191.75**	**3**	**60.29**	**<.001**
Search platform:Browser	0.20	6	49.08	.977
Keyword complexity:Browser	0.00	2	19.46	1.000
Search platform:Cache	0.62	3	60.29	.556
Keyword complexity:Cache	0.09	1	28.7	.743
Browser:Cache	0.07	2	19.46	.915
Search platform:Keyword complexity:Browser	0.19	6	49.08	.974
Search platform:Keyword complexity:Cache	0.65	3	60.29	.547
Search platform:Browser:Cache	0.13	6	49.08	.992
Keyword complexity:Browser:Cache	0.03	2	19.46	.972
Search platform:Keyword complexity:Browser:Cache	0.11	6	49.08	.995

Note

Significant (*p* < .05) relationships are highlighted with bold font.

**FIGURE 2:**
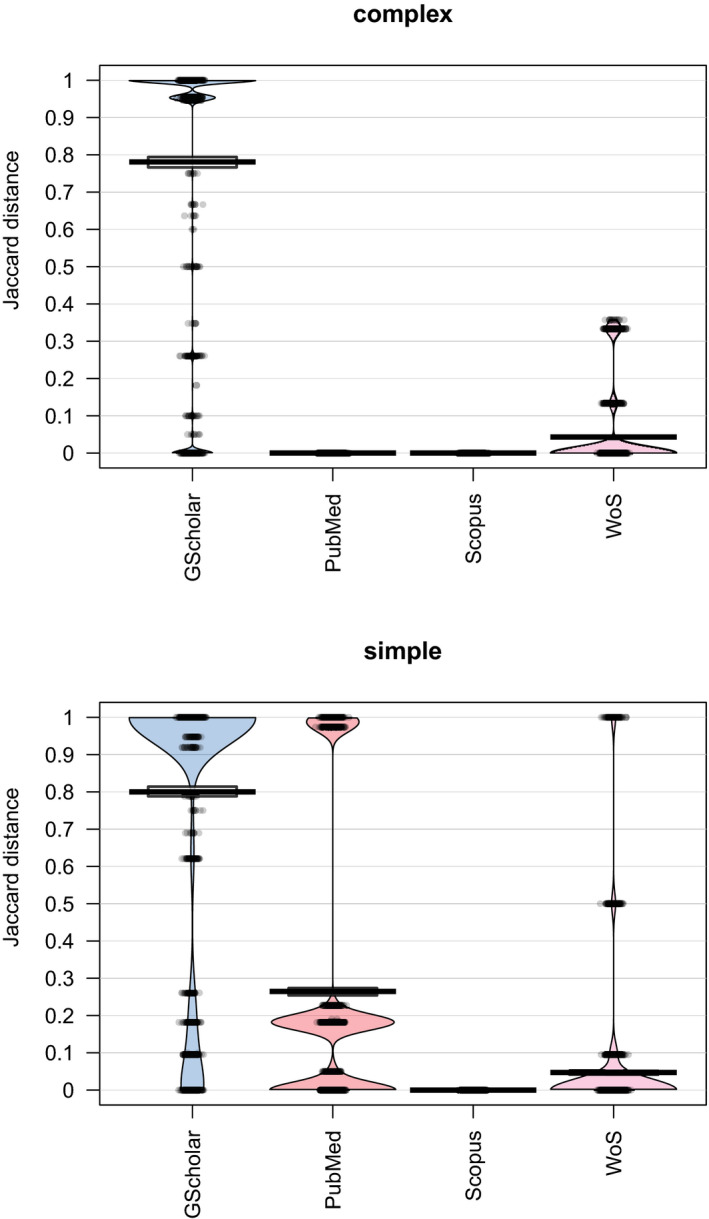
Pirate plots showing the average similarities of the first twenty papers within each search platform–keyword complexity group, for each search platform. Similarities were calculated based on binary matrices, using Jaccard distances. Median similarities are indicated with a thick black line, and grey circles are the data points. The outline of the diagram indicates the distribution of the data. Google Scholar is abbreviated as GScholar

The multivariate analysis run on the first twenty papers collected from each search revealed significant differences among the search platforms (dbRDA, bootstrapped *p*‐value = .01) but did not show a significant influence on browser choice or cache state. Areas of convex hulls defined by these ‘paper‐communities’ (see *Methods*) of the first twenty hits were zero for Scopus and for complex keyword searches in PubMed and Web of Science. Convex hull areas were the largest for Google Scholar (322.24, 491.90 for simple and complex keywords, respectively) and low (8.82) for simple keyword searches in Web of Science. When PubMed and Web of Science datasets were split by their update time, hulls for both PubMed subsets became zero but remained greater than zero for Web of Science. Jaccard distances showed a similar pattern; they were zero for Scopus, indicating no difference between the first twenty papers, and deviated from zero for all other platforms (Figure 2). After correcting for the database update, only Web of Science and Google Scholar hulls remained significantly greater than zero.

## 4 DISCUSSION

Here, we identified a shortcoming of scientific search platforms that can decrease the transparency and repeatability of the synthesis of quantitative evidence synthesis relying on database searches. This has a broad importance in the repeatability of systematic reviews and the reliability of the conclusions drawn.

Significant differences were evident in search platform consistency in terms of both the number of hits (the size of the body of available evidence) and its composition when identical search terms were queried from different institutions at different locations. We found that PubMed and Scopus had high consistencies, whilst Google Scholar and Web of Science were not consistent in the number of hits they returned. Google Scholar provided the greatest number of hits for every search but was the least consistent, though the composition of the evidence collected, characterized by the first twenty papers it returned, was relatively consistent. Web of Science also showed similarly low consistency in terms of numbers of hits returned from identical searches initiated from different locations/host institutions. Hit numbers and the returned list of articles from Scopus searches were consistent. PubMed varied in hit numbers and had great dissimilarities among the returned sets of papers, especially in those related to more general searches that necessarily had more hits. These dissimilarities were likely due to a database update that happened during our search exercise. Indeed, data showed that six papers for the simple ecology terms were added to the database during the course of this worldwide exercise. Since the papers listed were ordered according to their time of inclusion in the dataset, the first 20 collected papers would greatly differ and especially the larger values in the newly added articles can cause a disproportionally large effect on the similarity of the 20 collected papers. Once the differences before and after database update were accounted for, PubMed showed no deviation either in the number of returned papers or the list of the first 20 listed papers. A similar change in the dataset happened with Web of Science during our search, but differences remained even after correcting for the update. This suggests that discrepancies were caused by other sources, such as institute's location where the search was initiated from, which, in turn, suggests that differences in the institutional licenses to Web of Science–related services may cause experienced discrepancies in search results. Indeed, even the “Core Collection” of Web of Science, which we queried in our study, consists of several databases that may fall under different licenses in institutional subscriptions (Gusenbauer & Haddaway, 2019; Liu, 2019). On the other hand, Google Scholar is likely to be similar to the main Google search in its geographical dependencies when providing search result (Cooper et al., 2021). Overall, in our tests, Scopus and PubMed proved to be the most consistent databases, and Web of Science and Google Scholar produced less consistent results.

Although we could not thoroughly decipher the influence of browser or cache on the search results, there was an indication that these factors only affected Google Scholar outcomes. Google Scholar is known to optimize search hits according to the search history of its users; thus, even the differences between browsers are likely to be the results of participants’ previous browser use and, therefore, different cache contents in different browsers.

While the disadvantages of the inconsistencies in Google Scholar search results have been repeatedly illustrated (Jacsó, 2005, 2008), the similar behavior from Web of Science has only recently been reported (Gusenbauer & Haddaway, 2019) but in neither case was the variability estimated nor were the potential solutions we present below discussed. Given the widespread use of Web of Science, neglecting this discrepancy can mislead scientists when drawing conclusions from their evidence synthesis, when the body of evidence was collected by Web of Science searches alone. The use of only one database is generally discouraged (Higgins et al., 2019), and, although some authors mainly target Google Scholar‐based reviews (Haddaway et al., 2015; Jacsó, 2008), it is clear here that relying on Web of Science alone, or another single source, may lead to missing data or can make data‐synthesis studies irreproducible. Despite the recommendations to use multiple sources for such studies (see the PRISMA statement (Moher et al., 2009)), our rapid pilot search of 20 recent systematic reviews in leading journals showed that eight papers used only Web of Science (Appendix S1). Considering the concerns that using inadequate databases/search engines makes systematic reviews unreliable, it may be good for authors to clearly justify their search platform choice.

To improve the replicability of a systematic review we suggest the following points:

1. Researchers conducting systematic reviews should be aware of this problem and be explicit about the methodology they use to ensure sufficient consistency and repeatability (Rethlefsen et al., 2021). A detailed description should include the search platform used, the exact database used if search platform covers multiple databases, search date and time, the exact search strings, as well as whether the same search was replicated by more than one person. The locality/institution network from which the search was conducted should also be reported, preferably along with the IP address of the computer the queries were initiated from. Since even Web of Science's Core Collection consists of several sister databases, the precise reporting of the queried database should become common practice (Liu, 2019). The exact time of the search or the time window of the query are also essential. The holdings of databases, however, are not constant, historical records can be added over time, and, therefore, queries even within a clearly limited time period can deliver different result sets. Thus, reporting the time window of the queries can provide only a partial solution.
2. The use of adequate search platforms for a particular task should be an important consideration. All of the large platforms have different strengths; Google Scholar searches grey literature, Web of Science has the largest (combined) dataset, and, as our study confirmed, that Scopus and PubMed are the most consistent. Moreover, some databases may be more suitable for collecting information on a particular topic or have a greater historical coverage than others (Falagas et al., 2007). In some countries, local search engines/databases may perform well for multiple criteria (e.g. Nuñez & Amano, 2021).
3. Peer reviewers and journal editors have an important role in safeguarding the repeatability reviews by enforcing precise reporting according to already established criteria.
4. Providers of scientific search platforms should consider opening their search code and relaxing their paywalls to make the full list of references resulted from a search publicly available (Shotton, 2018), thus contributing to search transparency and, hence, scientific repeatability. Particularly Web of Science, as probably the most commonly used search platform, should act on making its search hits equally reachable to all users and, rather than a priori filtering them according to the institutions’ paywall, restrict access only *after* the primary result set has been provided to the user.
5. Since Google Scholar has been criticized by the scientific community for the obscurity of its search algorithms (van Dijck, 2010), it could increase transparency in this regard to allow researchers to understand how the hit results are generated and how these are ordered. We acknowledge the business imperative but the need for research rigor is an important public good and facilitating this would enhance social license.
6. Providing well‐documented, standard application programming interfaces (APIs) would be greatly beneficial for researchers. These APIs could generate unique identifiers for searches and combine search term, result list, search time and location, and additional metadata (e.g. computing environment). Using an API for standardized searches would be particularly beneficial for searches using Google Scholar that shows a strong dependence on the computing environment. Although this solution could control for a great deal of variation derived mostly from computing background and would be able to keep detailed records on the metadata of the searches, it also brings up novel challenges. Firstly, APIs are admittedly more complex in terms of functionality and also in their use (which often needs some programming knowledge) than simple web interfaces. These may discourage users. Moreover, collecting detailed data about search locations, or even computing environment, raises both security and privacy concerns. Finally, storing individual searches along with the necessary metadata may be resource heavy, which is likely to increase maintenance costs, and therefore the subscription fees, of these services.
7. Alternatively, systematic review authors could deposit full list of their retrieved papers in open repositories as it is often done with raw data in many research areas. Alongside of these search outputs, metadata in a standard (machine readable) format about the search environment could be saved and deposited in these repositories. Web of Science, for instance, allows users to save search histories in *.wos files which, beside the search term, contain the exact queried databases. More studies are needed to confirm if using restricted databases provides a higher consistency in hit results among institutions.

Despite the limited number of institutions that participated in this exercise, with an overrepresentation of European locations, and the lack of contribution from African, South American, and other Asian countries, we found, even within the European countries, variation among the numbers of search hits. This suggests that adding more countries would have led to even greater variability in the resulting datasets. It could be valuable to test a wider range of search platforms and subjects to gain further understanding of the level of reliability of various systems and test their strengths and weaknesses.

Should the above steps towards ensuring repeatability not happen, the criticism of systematic reviews will grow (Ioannidis, 2016) and the power of this approach to handle contentious issues with a reliable evidence base (Higgins et al., 2019) may be eroded. The appearance of automatically generated systematic reviews, relying on artificial intelligence (Beller et al., 2018) are likely to exacerbate the problem. Although repeatable searches will not solve all the current systematic review problems, such as poor reporting of the methods or other transparency issues, they are an important step to make systematic review repeatable and thus synthetize scientific knowledge objectively.

We conclude that in order to ensure repeatability of scientific searches, search platforms, particularly those pertinent for systematic reviews, should collaborate with researchers. Since raw data input can significantly influence the output of a study and, in the age of big data, studies on published results are becoming more common, an unbiased and timely way of data extraction is needed, for example through automatized APIs. At present, updating systematic reviews using precisely repeated methodology is problematic (Garner et al., 2016); hence a clear decision map on the advantages and disadvantages of particular databases and search engines should be drawn to ensure the integrity of publication‐based studies.

## CONFLICT OF INTEREST

The authors declare no conflict of interest.

## AUTHOR CONTRIBUTIONS

**Gabor Pozsgai:** Conceptualization (lead); Data curation (lead); Formal analysis (lead); Investigation (lead); Methodology (lead); Project administration (equal); Software (lead); Validation (lead); Visualization (lead); Writing‐original draft (lead); Writing‐review & editing (lead). **Gabor L. Lövei:** Conceptualization (equal); Data curation (supporting); Investigation (equal); Methodology (equal); Project administration (equal); Resources (equal); Supervision (lead); Validation (equal); Writing‐review & editing (lead). **Liette Vasseur:** Data curation (supporting); Formal analysis (supporting); Investigation (equal); Methodology (equal); Project administration (equal); Resources (equal); Supervision (equal); Validation (equal); Writing‐review & editing (equal). **Geoff Gurr:** Conceptualization (equal); Data curation (supporting); Investigation (equal); Resources (lead); Software (equal); Supervision (equal); Validation (equal); Writing‐review & editing (equal). **Péter Batáry:** Data curation (supporting); Investigation (supporting); Methodology (supporting); Resources (supporting); Validation (supporting); Writing‐review & editing (equal). **Janos Korponai:** Data curation (supporting); Investigation (supporting); Methodology (supporting); Resources (supporting); Validation (supporting); Writing‐review & editing (supporting). **Nick A. Littlewood:** Data curation (supporting); Investigation (supporting); Resources (supporting); Validation (supporting); Writing‐review & editing (supporting). **Jian Liu:** Data curation (supporting); Investigation (supporting); Resources (supporting); Validation (supporting); Writing‐review & editing (supporting). **Arnold Móra:** Data curation (supporting); Investigation (supporting); Resources (supporting); Validation (supporting); Writing‐review & editing (supporting). **John Obrycki:** Data curation (supporting); Investigation (supporting); Resources (supporting); Validation (supporting); Writing‐review & editing (supporting). **Olivia Reynolds:** Data curation (supporting); Investigation (equal); Resources (supporting); Validation (supporting); Writing‐review & editing (equal). **Jenni A. Stockan:** Data curation (supporting); Investigation (supporting); Resources (supporting); Validation (supporting); Writing‐review & editing (supporting). **Heather VanVolkenburg:** Data curation (supporting); Investigation (supporting); Resources (supporting); Validation (supporting); Writing‐review & editing (supporting). **Jie Zhang:** Data curation (supporting); Investigation (equal); Validation (supporting); Writing‐review & editing (supporting). **Wenwu Zhou:** Data curation (supporting); Investigation (equal); Resources (supporting); Validation (supporting); Writing‐review & editing (supporting). **Minsheng You:** Funding acquisition (lead); Project administration (equal); Resources (lead); Supervision (lead); Validation (equal); Writing‐review & editing (lead).

## Supporting information

Appendix S1

Appendix S2

Appendix S3

Appendix S4

Appendix S5

## Data Availability

All data are deposited in the Dryad Digital Repository (https://doi.org/10.5061/dryad.djh9w0w17). The computer script associated with this project is hosted on GitHub code repository (https://github.com/pozsgaig/search_location).
