# Supplementary figures and images for: Irreproducibility in searches of scientific literature: A comparative analysis
Source: Ecol Evol. 2021 Oct 14;11(21):14658–68. doi: 10.1002/ece3.8154 (PMC8571571; doi:10.1002/ece3.8154)

### Scaled deviation from the mean number of hits per group

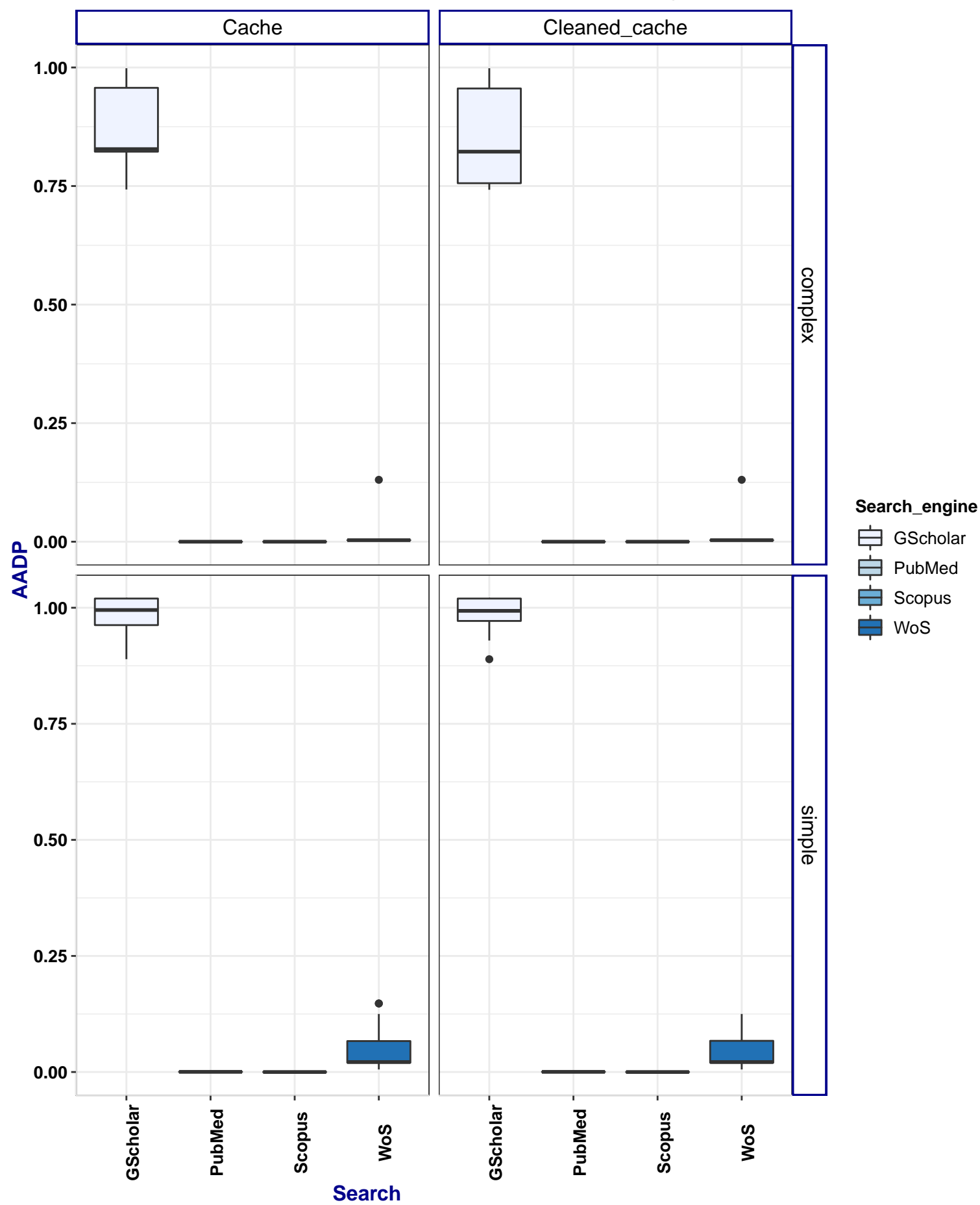

Supplement: Supplementary file 4 — Appendix S4 [file ECE3-11-14658-s002.pdf]

**Scaled deviation from the mean number of hits per group**

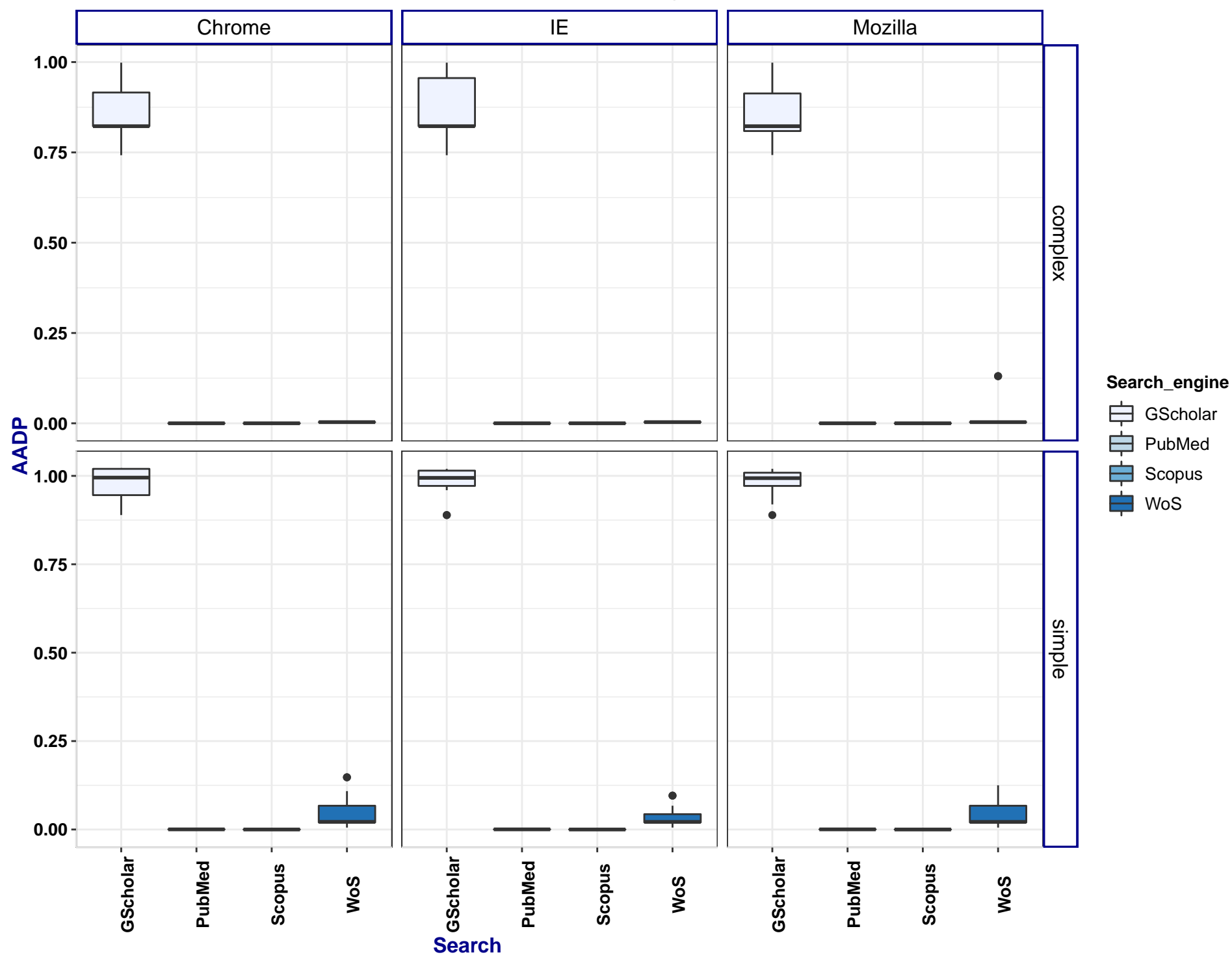

Supplement: Supplementary file 5 — Appendix S5 [file ECE3-11-14658-s005.pdf]
